# Isolation and Characterization of Small Extracellular Vesicles from Porcine Blood Plasma, Cerebrospinal Fluid, and Seminal Plasma

**DOI:** 10.3390/proteomes7020017

**Published:** 2019-04-25

**Authors:** Helena Kupcova Skalnikova, Bozena Bohuslavova, Karolina Turnovcova, Jana Juhasova, Stefan Juhas, Marie Rodinova, Petr Vodicka

**Affiliations:** 1 Institute of Animal Physiology and Genetics of the Czech Academy of Sciences, Rumburska 89, 27721 Libechov, Czech Republic; bohuslavova@iapg.cas.cz (B.B.); karolina.turnovcova@iem.cas.cz (K.T.); juhasova@iapg.cas.cz (J.J.); juhas@iapg.cas.cz (S.J.); marie.rodinova@lf1.cuni.cz (M.R.); vodicka@iapg.cas.cz (P.V.); 2 Institute of Experimental Medicine of the Czech Academy of Sciences, Videnska 1083, 14220 Prague, Czech Republic; 3 Department of Pediatrics and Adolescent Medicine, First Faculty of Medicine, Charles University and General University Hospital in Prague, Ke Karlovu 2, 12109 Prague, Czech Republic

**Keywords:** extracellular vesicle, exosome, body fluid, plasma, cerebrospinal fluid, seminal plasma, pig model, proteomics

## Abstract

Extracellular vesicles (EVs) are a highly attractive subject of biomedical research as possible carriers of nucleic acid and protein biomarkers. EVs released to body fluids enable indirect access to inner organs by so-called “liquid biopsies”. Obtaining a high-quality EV sample with minimum contaminants is crucial for proteomic analyses using LC–MS/MS or other techniques. However, the EV content in various body fluids largely differs, which may hamper subsequent analyses. Here, we present a comparison of extracellular vesicle yields from blood plasma, cerebrospinal fluid, and seminal plasma using an experimental pig model. Pigs are widely used in biomedical research as large animal models with anatomy and physiology close to those of humans and enable studies (e.g., of the nervous system) that are unfeasible in humans. EVs were isolated from body fluids by differential centrifugation followed by ultracentrifugation. EVs were characterized according to protein yields and to the quality of the isolated vesicles (e.g., size distribution, morphology, positivity for exosome markers). In our experimental setting, substantial differences in EV amounts were identified among body fluids, with the seminal plasma being the richest EV source. The yields of pellet proteins from ultracentrifugation of 1 mL of porcine body fluids may help to estimate body fluid input volumes to obtain sufficient samples for subsequent proteomic analyses.

## 1. Introduction

Extracellular vesicles (EVs) are membrane-enveloped particles released by cells to surrounding tissue microenvironment and body fluids. According to their origin, apoptotic bodies, microvesicles, and exosomes are recognized as the major types of EVs. Because the origin is difficult to assess in already released EVs, the term “small EVs” has been suggested for vesicles below 200 nm by the International Society for Extracellular Vesicles [1]. Small EVs contain molecules of EV-producing cells, which are incorporated into the vesicles during their biogenesis [2]. The lipid composition of the small EV bilayer membrane is distinct from that of the plasma membrane of the source cell, as the EV membrane is enriched in detergent-resistant lipid rafts. The EV internal cargo consists mainly of proteins, nucleic acids, and small molecules [2,3]. Proteins with targeted incorporation into EVs during their biogenesis may be used as EV markers. For example, proteins Alix, TSG101, CD63, and CD9 are enriched in exosomes and commonly used as markers of exosomes [3]. According to the current proteomic studies, EV protein composition varies by the cell of origin. 

EVs and, particularly, exosomes are recognized as particles that are able to transfer information in the form of proteins, nucleic acids, lipids, or sugars to recipient cells [4,5] and thus play a significant role in intercellular communication. As EVs are accessible from body fluids (concept of “liquid biopsy”), they are an object of intensive investigation as a possible source of biomarkers of various diseases, including cancer [6], neurodegeneration [7,8], cardiovascular diseases [9], and others. EVs have also high potential in therapy, both as native EVs, directly used as therapeutic agents in tissue regeneration and immunomodulation, and in a form of engineered EVs to deliver biologically active material to target cells [10,11,12]. 

Pigs not only are economically important farm animals used for food production but also represent a valuable biomedical model to study a plethora of human diseases and develop therapies. Pigs share anatomical, physiological, and pathophysiological similarities with humans [13] and also allow the production of genetically modified models for translational research [14]. Miniature pigs have an adult body size of approximately 80 kg and enable the use of identical instrumentation and therapy doses as in human patients. Our institute breeds various minipig models including models of neurodegenerative Huntington’s disease [15], spinal cord injury [16], and melanoma [17]. EVs in pig body fluids may represent a potential source of molecules to study physiological and pathological processes. 

Blood is an easily accessible and frequently analyzed body fluid in both human and veterinary medicine. Blood flows through the whole organism and contains products of various cell types. However, EV isolation from blood plasma for biomarker discovery faces several obstacles. The majority of circulating blood EVs originates from blood cells themselves, with the platelet-derived EVs released during platelet activation being the predominant fraction [18]. The percentage of the desired EV subtype may be very low [19], and the EV content in blood is further influenced by blood collection and processing procedure (see [20] for details). Nonetheless, tissue-specific EVs can be isolated from blood plasma by immunocapturing, using specific antibodies recognizing EV surface molecules, such as NCAM or L1CAM for neural cell-derived EVs [6,21,22], EpCAM for epithelial cells including breast or ovarian cancer-derived EVs [23], or prostate-specific membrane antigen (PSMA) for prostate cell-derived EVs [24,25]. The estimation of EV content in human plasma largely varies in different studies according to the isolation and detection techniques, with a mean of about 10^10^ particles per mL of plasma [26]. Blood plasma EVs are most frequently studied in the context of cancer biomarkers, and elevated absolute numbers of plasma EVs were found in several cancers [26].

Cerebrospinal fluid (CSF) is a biological fluid that surrounds the brain and spinal cord and provides mechanical support and protection to the central nervous system (CNS). CSF is produced by ultrafiltration of blood plasma by ependymal cells in the choroid plexuses of the brain ventricles [27]. The protein content in CSF is 100 to 300 times lower than in blood plasma, demonstrating a high efficacy of the blood–CSF barrier [28]. Approximately 80% of CSF proteins originate from the blood, while only 20% of proteins are released from nervous cells [28]. As the CSF is in direct contact with the central nervous system, EVs in CSF are studied as a potential source of biomarkers of neurodegeneration [29,30], inflammation [31], ischemia/stroke [32,33], or tumors [34,35]. Nonetheless, the collection of CSF requires an invasive procedure with risk of complications (e.g., bleeding, infection). Several studies have suggested that EVs may cross the blood–brain barrier (BBB), and neural cell-derived EVs may be detectable in blood plasma. The exact mechanism of BBB crossing is not yet known [7,36]. EVs are also supposed to participate in the spreading of misfolded proteins among cells in neurodegenerative diseases [37].

Seminal plasma is produced mainly by accessory sex glands of the male genital system. Seminal plasma is rich in vesicles, predominantly prostasomes produced by epithelial cells of the prostate [38]. Seminal plasma EVs are crucial for sperm maturation, prevention of premature acrosome reaction, prevention of premature capacitation, and for successful fertilization [39]. Depletion of EVs from boar seminal plasma leads to decreased sperm motility, shorter survival time, and declined sperm plasma membrane integrity [40]. The collection of semen requires a non-invasive procedure. In boars, mean volumes of 250 mL of semen can be collected. The first 50 mL of semen are richest in sperms. The long-term interest of our institute in reproduction, in vitro fertilization, as well as decline of fertility and testes pathology studied in boars transgenic for a mutant human huntingtin gene [41], places semen at center stage in our investigations. In human, seminal plasma-derived EVs are mostly studied in relation to male fertility but have potential also in prostate cancer research [18].

As the populations of EVs isolated from body fluids and conditioned culture media are mostly heterogenous, the International Society of Extracellular Vesicles proposed guidelines for EV characterization before their usage in molecular or functional studies [1]. 

The aim of this study was to compare small EV amounts in porcine blood plasma, CSF, and seminal plasma and evaluate these body fluids as possible sources of small EVs for future proteomic biomarker discovery studies. Special attention was paid to quantity and quality of the isolated EVs, regarding their total protein content, size, morphology, and positivity for EV markers.

## 2. Materials and Methods 

### 2.1. Experimental Animals

Twenty adult miniature pigs of Göttingen and Minnesota mixed genetic background [15] were employed in this study. Females were housed in groups of 3–4, boars were kept individually. Blood plasma and CSF were collected from animals of both sexes of 1–3 years of age. Semen was collected from boars of 6–7 years of age. All experiments were carried out according to the guidelines for the care and use of experimental animals and approved by the Resort Professional Commission of the Czech Academy of Sciences for Approval of Projects of Experiments on Animals (Approved protocols No. 53/2015 and 75/2017).

### 2.2. Isolation of EVs from Blood Plasma

EV isolation from blood plasma was performed according to Théry et al. [42] with modifications by recent protocols [43,44,45,46]. Venous blood (30 mL) was collected from animals starved for 12 h by venipuncture of vena cava cranialis by a 20G needle. An isotonic sodium citrate pH 7.4 (final concentration 0.5%) was used as an anticoagulant. Blood was immediately centrifuged at 2500 g for 15 min at room temperature to remove blood cells and prevent platelet activation and release of platelet-derived EVs. Supernatant was transferred into a new tube (bottom 10% of the supernatant above the blood cells were discarded) and centrifuged again at 2500 g for 15 min at room temperature. Supernatant (platelet-poor plasma, approximate volume 15 mL) was cooled to 4 °C, and all the following steps were carried out at 4 °C. Platelet-poor plasma was diluted 1:1 by 0.22 µm filtered ice-cold PBS to decrease sample viscosity and centrifuged in 50 mL polypropylene bottles at 10,000 g for 35 min at 4 °C (Avanti J centrifuge, JS13.1 rotor, k-factor 1841, Beckman Coulter, Indianapolis, IN, USA). Supernatant was processed by ultracentrifugation in UltraClear 17 mL tubes, 130 min at 4 °C and 28,000 rpm, corresponding to 100,000 g at r_avg_ (Optima L-90K ultracentrifuge, SW32.1 rotor, k-factor 229, Beckman Coulter, Indianapolis, IN, USA). Pellet was resuspended in filtered ice-cold PBS and centrifuged again at 28,000 rpm for 70 min at 4 °C using the same tubes and rotor. The final pellet containing EVs was resuspended in 150 µL PBS, aliquots were directly used for flow cytometry and grid preparation for electron microscopy. The other part of the resuspended pellet was stored at −80 °C and used later for protein extraction.

All blood samples were tested for normal blood cell counts (red blood cells, white blood cells, differential white blood cell count, platelets) using a hematologic analyzer ADVIA 120/2120/ 2120i (Siemens Healthineers, Erlangen, Germany) operated according to the manufacturer´s instructions.

### 2.3. Isolation of EVs from Cerebrospinal Fluid

EVs were isolated from CSF using adapted published protocols [47,48,49]. Cerebrospinal fluid was obtained by lumbar puncture in 12 h starved animals, anesthetized with a KTX mixture (ketamine, tiletamine, xylazine, 1 mL per 15 kg of body weight). After cleaning and disinfection of the lumbar area, 5–8 mL of CSF per animal were collected using a spinal needle. The puncture area was covered by a liquid dressing, and the animal was let to recover. CSF samples were immediately centrifuged at 2000 g for 10 min at 4 °C and carefully inspected for the absence of a red blood cell pellet. After additional centrifugation at 2400 g 10 min at 4 °C, the supernatant was directly used for EV isolation or stored at −80 °C. CSF samples from several animals were pooled to reach a final volume of at least 18 mL. Extracellular vesicles were isolated by ultracentrifugation of CSF in UltraClear 17 mL tubes, 70 min at 4 °C and 28,000 rpm, corresponding to 100,000 g at r_avg_ (Optima L-90K ultracentrifuge, SW32.1 rotor, k-factor 229, Beckman Coulter, Indianapolis, IN, USA). Pellet was resuspended in 0.22 µm filtered ice-cold PBS and ultracentrifuged using the same settings. The final pellet containing EVs was resuspended in 100 µL PBS, aliquots were directly used for flow cytometry and grid preparation for electron microscopy. The other part of the resuspended pellet was stored at −80 °C and used later for protein extraction. 

### 2.4. Isolation of EVs from Seminal Plasma

Boar semen collection and processing for EV isolation was performed according to modified published protocols [40,50,51,52]. Boar semen was collected by gloved-hand technique. Sperm-rich fraction, i.e., first 50 mL of ejaculate, were collected and kept at 37 °C for a maximum of 1 h. Semen was filtered through a sterile filter paper to remove the gelatinous secrete of Cowper´s glands. All semen samples were evaluated using the Microptic (Barcelona, Spain) sperm cell analyzer to assess the number of spermatozoa, their motility, and progressivity. All samples used for EV isolation had physiologic sperm cell values. 

Semen was centrifuged 15 min at 800 g, 4 °C, to pellet sperms. Supernatant (seminal plasma, 30–35 mL) was transferred to a new tube and centrifuged 30 min at 3000 g and 4 °C (Allegra X-22R centrifuge, SX4250 rotor, Beckman Coulter, Indianapolis, IN, USA). The 3000 g supernatant (25–30 mL) was further spun at 10,000 g in 50 mL polypropylene bottles for 35 min at 4 °C (Avanti J centrifuge, JS13.1 rotor, k-factor 1841, Beckman Coulter, Indianapolis, IN, USA). Supernatant (17 mL) was processed by ultracentrifugation in UltraClear tubes, 70 min at 4 °C and 28,000 rpm, corresponding to 100,000 g at r_avg_ (Optima L-90K ultracentrifuge, SW32.1 rotor, k-factor 229, Beckman Coulter, Indianapolis, IN, USA). Pellet was resuspended in filtered ice-cold PBS and ultracentrifuged again using the same settings. The final pellet containing EVs was resuspended in 500 µL of PBS, aliquots were directly used for flow cytometry and grid preparation for electron microscopy. The other part of the resuspended pellet was aliquoted, stored at −80 °C, and used later for protein extraction. 

### 2.5. Flow Cytometry

Aliquots of ultracentrifuged pellets resuspended in PBS were diluted to 500 µl with filtered PBS and labeled with 5(6)-Carboxyfluorescein diacetate N-succinimidyl ester (CFSE, eBioscience) according to the manufacturer´s instructions. Briefly, CFSE was added to the samples to reach 10 µM final concentration in labeling solution, and all samples, including labeled PBS buffer used for EV isolation (background sample), were incubated 60 min in a 37 °C water bath. The labeling reaction was stopped with 1 µl fetal bovine serum, and all samples, including calibration beads (ApogeeMix, Apogee Flow Systems, Hemel Hempstead, Hertfordshire, UK), unlabeled buffer, CFSE-labeled buffer, unlabeled EVs, and CFSE-labeled EV samples were analyzed by the Apogee A50-Micro flow cytometer (Apogee Flow Systems). Using calibration beads, small particles were then categorized to 110, 180, 240, 300, 500, 880 and 1300 nm classes, and the number of CFSE-positive events per µl was counted in each class. The background signal (CFSE-labeled PBS buffer) was subtracted from particle counts, and the number of small particles was then calculated for the entry amount of 1 mL of each body fluid sample.

### 2.6. Electron Microscopy

Five microliters of 100,000 g pellets resuspended in PBS was incubated on formvar/carbon-coated 400 mesh copper grids for 20 min, protected from dust. The grids were then incubated with a droplet of 2% formaldehyde in PBS for 20 min, followed by 6x3 min washes with droplets of ultrapure water. Grids were contrasted for 12 min in 2% uranylacetate on ice in the dark, washed in water, and let to dry. Images were acquired on a transmission electron microscope JEOL 1011 (Tokyo, Japan) equipped by a Veleta CCD camera and Olympus Soft Imaging Solution acquisition software.

### 2.7. Protein Extraction and Western Blotting

The 100,000 g pellets resuspended in PBS were mixed with equal volumes of 2x concentrated ice cold RIPA buffer (final concentration in lysates 150 mM NaCl, 5mM EDTA, 50 mM Tris HCl, 0.5% NP-40, 1% sodium deoxycholate, 1% Triton X-100, 0.1% SDS at pH 7.4, and 1x concentrated HALT protease and phosphatase inhibitor) and incubated for 10 min on ice with occasional vortexing. The lysates were sonicated and centrifuged at 16,000 g for 10 min to remove unlysed debris. Protein concentration in the supernatants was determined by BCA protein assay. The original body fluids were processed in the same way, the amount of RIPA lysis buffer for sample dilution was adjusted to achieve 0.5–3 mg/mL protein concentration in lysates.

Two micrograms of protein extract were loaded on precast 4–12% polyacrylamide minigels and separated for 90 min at 150 V. The proteins were transferred from the gels to nitrocellulose membranes using iBlot (Thermo Fisher Scientific, Waltham, MA, USA) semidry system. Membranes were blocked in 1% or 5% skimmed milk or 5% bovine serum albumin (BSA) in TTBS (tris-buffered saline with 0.05% Tween20) according to primary antibody dilution. Anti-Alix 1:1500 (Abcam, ab88388, Cambridge, UK), anti-TSG101 1:500 (Origene, TA333922, Rockville, MD, USA), anti-β-tubulin 1:2000 (Merck, T4026, Kenilworth, NJ, USA), anti-UQCRC1 1:250 (Merck, HPA002815, Kenilworth, NJ, USA), anti-CD63 1:1000 (Santa Cruz Biotechnology, sc5275, Dallas, TX, USA), antibodies were diluted in 5% milk in TTBS, anti-lamin A/C 1:2000 (Merck, SAB4200236, Kenilworth, USA) in 1% milk, and anti-CD9 1:500 (Aviva, ARP61171, London, UK), anti-CD9 1:20 (Sysmex, CP638591, Kobe, Japan), and anti-CD63 1:200 (Exbio, clone MEM-259, Prague, Czech Republic) in 5% BSA, followed by peroxidase-conjugated secondary antibodies (anti-rabbit or anti-mouse IgG, Jackson ImmunoResearch 711-035-152 and 715-035-151, resp., as appropriate, West Grove, PA, USA) used for specific protein detection. In addition, peroxidase-conjugated anti-swine-IgG (Jackson ImmunoResearch 114-035-003, West Grove, PA, USA) was used for direct detection of immunoglobulin chains in plasma samples. After incubation of the membranes with ECL Prime chemiluminescent substrate, images were acquired on Chemidoc XRS imaging system (Bio-Rad, Hercules, CA, USA). Pig brain and testes (from animals sacrificed in previous experiments) and human Neural Stem Cells (H9-Derived) RIPA extracts were used as positive controls for western blots. After ECL detection, the membranes were washed in ultrapure water and incubated for 5 min in silver staining solution (1 g sodium citrate, 0.4 g FeSO_4_, 0.1 g AgNO_3_, ultrapure water up to 50 mL). The membranes were intensively washed in ultrapure water to stop the staining reaction and scanned on a Chemidoc XRS imaging system.

## 3. Results and Discussion

**Figure 1:**
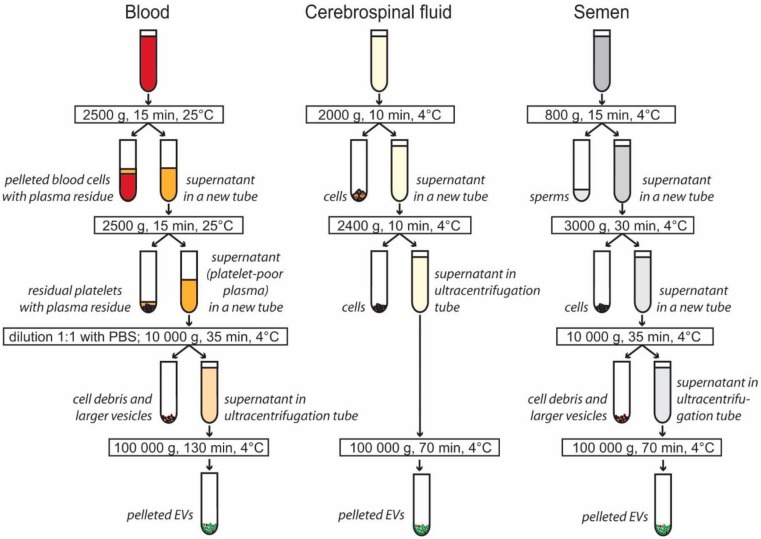
Centrifugation steps used to isolate extracellular vesicles (EVs) from blood plasma, cerebrospinal fluid, and semen. Differential centrifugation was used to pellet cells, cell debris, and larger extracellular vesicles. Small and medium EVs were pelleted by ultracentrifugation. The EV pellet was washed in PBS, and additional ultracentrifugation at 100,000 g for 70 min was applied to all samples (not shown in figure).

EV isolation by differential centrifugation and ultracentrifugation (Figure 1) was chosen because of its broad usage and capacity to process large sample volumes. The time and g-force of individual centrifugation steps were carefully selected from currently available protocols to pellet small EVs (see methods for details). In the case of seminal plasma, a visible pellet was obtained after ultracentrifugation. Hardly visible pellets were obtained from both plasma and CSF. The centrifugation step at 10,000 g was not used for CSF samples to minimize EV loss.

**Figure 2:**
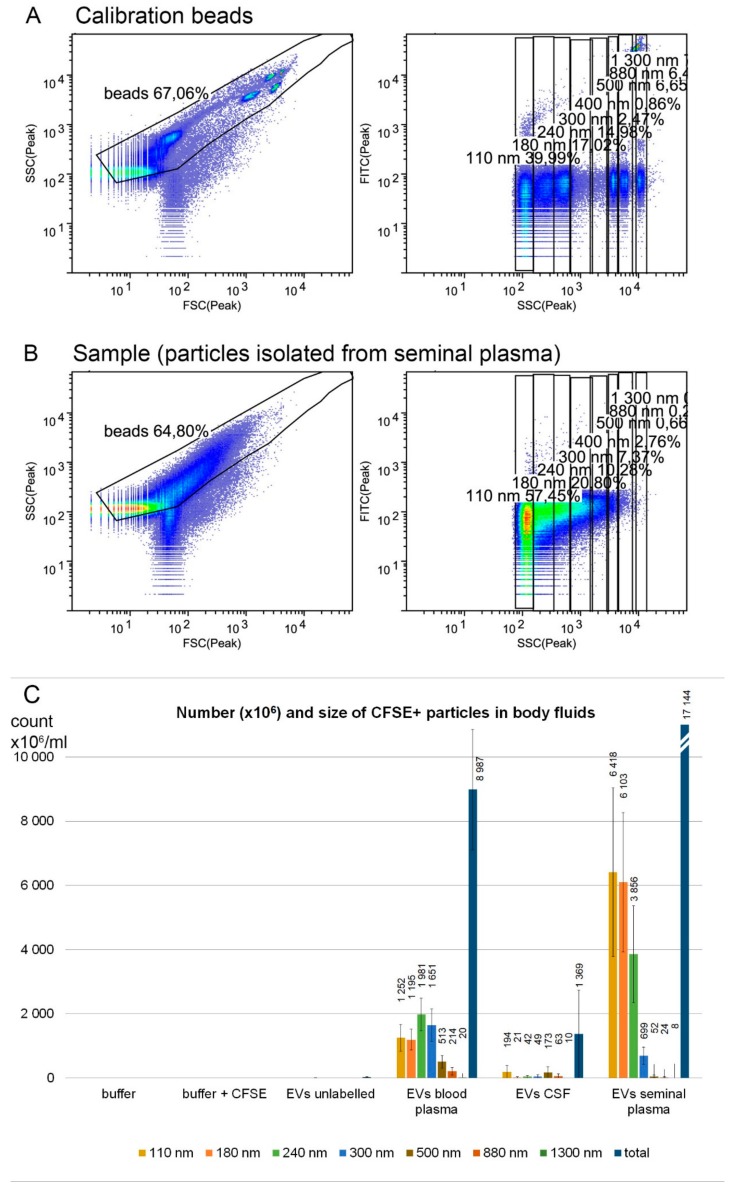
EV characterization by flow cytometry. A bead mixture (latex fluorescent beads at 110 and 500 nm, silica beads at 180, 240, 300, 590, 880, and 1300 nm) was used to calibrate the cytometer (**A**) and number and size of 5(6)-carboxyfluorescein diacetate N-succinimidyl ester (CFSE)-positive particles in body fluids were estimated (**B**). Ultracentrifugation pellets of blood plasma (n = 5), cerebrospinal fluid (n = 5), and seminal plasma (n = 8) were analyzed (**C**). The highest number of nanoparticles was detected in seminal plasma, followed by blood plasma and cerebrospinal fluid. The particle size was mostly below 300 nm, corresponding to small and medium EVs. Samples were labeled by CFSE fluorescent dye to enhance the detection of small particles in the flow cytometer. Particles below 110 nm might have been underestimated, as they are on the detection limit of the cytometer. Error bars represent standard errors of the mean.

To determine the size distribution of isolated particles as well as assess possible contamination by larger particles, Apogee Flow A50 micro cytometer capable of detection of particles in the range from approximately 100 nm to 20 µm was used. The CFSE fluorescent staining was used to improve the detection of small particles in the cytometer. The instrument was calibrated by a mixture of non-fluorescent silica beads and fluorescent latex beads with sizes from 110 nm to 1300 nm (Figure 2A). Silica reference beads have a refractive index close to that of biological particles. Predominantly, particles under 300 nm in diameter were isolated by our protocol from all three body fluids, corresponding in size to exosomes and small microvesicles (Figure 2B,C). The estimated numbers and size distribution of the particles for each body fluid are shown in Figure 2. The highest number of nanoparticles was detected in seminal plasma (on average, 1.7 × 10^10^ particles/mL of body fluid), followed by blood plasma (9 × 10^9^) and cerebrospinal fluid (1.4 × 10^9^).

**Figure 3:**
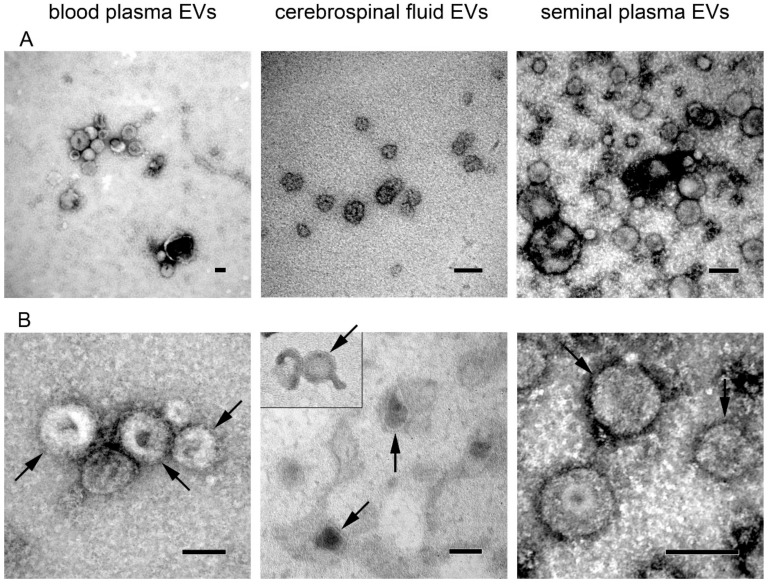
Transmission electron microscopy images of extracellular vesicles isolated from blood plasma, cerebrospinal fluid, and seminal plasma. (**A**) Wide-field images show size distribution of EVs (magnification 50,000–100,000×); (**B**) Close-up images showing vesicle morphology (magnification 150,000–250,000×). Bilayer membrane is highlighted by arrows. All scale bars represent 100 nm.

Size and morphology of EVs isolated from body fluids were assessed by transmission electron microscopy (Figure 3). Wide-field images confirmed the presence of cup-shaped vesicles whose size was mostly below 150–200 nm in all body fluids (Figure 3A). At a high magnification (200,000–250,000×), the bilayer membrane was detectable (Figure 3B, arrows) in EVs from all three body fluids. 

**Figure 4:**
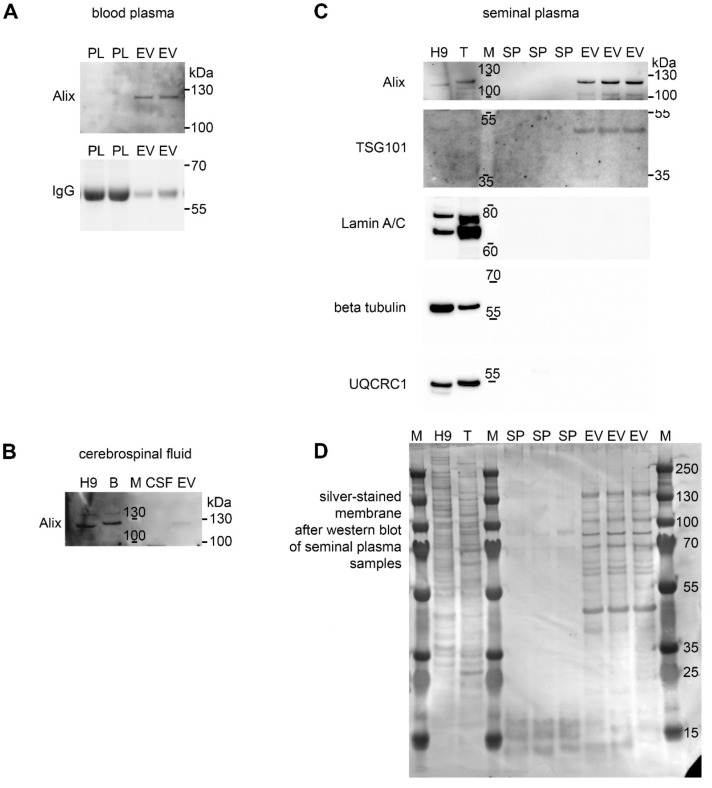
EV analysis by western blotting. Proteins from crude body fluids (blood plasma, PL, (**A**); cerebrospinal fluid, CSF, (**B**); seminal plasma, SP, (**C**) of individual animals as well as extracellular vesicles (EV) isolated from such biofluids were separated by SDS-PAGE and blotted to detect the EV markers Alix (110 kDa) and TSG101 (44 kDa). Porcine IgG heavy chains highly abundant in blood plasma were largely removed during EV isolation. The membrane was silver stained to detect protein loads in all lanes (**D**). Positive controls included human H9-derived neural stem cell line (H9) and porcine tissues (B = brain, T = testes). M = molecular weight standard. Representative images from at least three experiments are shown.

Western blotting was used to confirm the presence of the EV marker Alix in EVs enriched from all three porcine body fluids (Figure 4A–C). Despite the same loading amount of total proteins from ultracentrifugation pellets, the signal intensity for Alix varied among body fluids, with the strongest signal in seminal plasma and the weakest in CSF-derived ultracentrifugation pellets, suggesting different composition of vesicles of different origin. The Alix protein was undetectable in original body fluids used for EV isolation, which confirmed the successful enrichment of EVs by our isolation protocol. Membranes with plasma samples were reprobed with anti-pig IgG antibody, revealing a substantial removal of immunoglobulin G (a major blood plasma protein) heavy chains during the isolation of EV compared to the original blood plasma samples (Figure 4A). The seminal plasma EV samples, together with controls, were further analyzed for the presence of the exosomal marker TSG101 as well as of possible contaminants including nuclear (lamin A/C), cytoplasmic (β-tubulin), and mitochondrial (UQCRC1) proteins. Such major contaminants were undetectable in EVs (Figure 4C). After chemiluminescent detection of proteins, the membranes were silver stained to visualize protein loads in individual lanes. The presence of additional exosome markers from the tetraspanin family, i.e., CD63 and CD9, was analyzed in seminal plasma-derived EV; however, no cross-reactivity of antibodies with porcine tetraspanin proteins (even with positive controls) was detected, probably due to low homology with human proteins. Both reducing and non-reducing conditions in combination with polyvinylidene difluoride (PVDF) and nitrocellulose membranes were tested.

**Figure 5:**
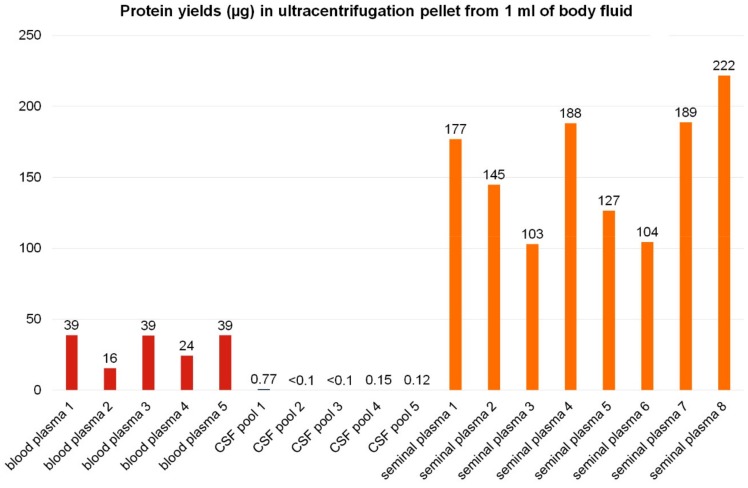
Yields of pellet proteins from ultracentrifugation of 1 mL of body fluids. Extracellular vesicles were enriched from body fluids by ultracentrifugation. The protein content in 100,000 g pellets in micrograms of protein isolated from 1 mL of individual samples is shown for blood plasma (red), cerebrospinal fluid (blue), and seminal plasma (orange). In the case of blood plasma and seminal plasma, each column represents an individual sample from one animal. CSF was pooled from several animals to obtain a sufficient volume for EV isolation.

The total protein content in the 100,000 g pellets substantially differed among individual body fluids (Figure 5). The ultracentrifugation pellets were lysed in RIPA buffer. The sample dilution in 1× RIPA buffer was optimized for each individual body fluid and body fluid ultracentrifugation pellets to ensure the maximal protein concentration in lysates below 3 mg/mL and proper protein extraction and solubilization and to minimize protein loss. The total protein content in the lysates was determined by BCA assay and normalized to 1 mL entry amount of body fluid to enable an easy comparison of EV protein yields among body fluids. 

From 1 mL of porcine blood plasma, the average yield of 31 µg of proteins per 100,000 g pellet was obtained. Such amount is in general sufficient for bottom-up proteomic analyses using LC–MS/MS systems. Nonetheless, the content of the EV proteins may be lower, as several other particles may co-isolate during EV enrichment by ultracentrifugation from blood plasma, including lipoproteins, viruses, and others [5,26]. Decreased Alix positivity in blood plasma pellets compared to seminal plasma may be caused by co-isolation of lipoproteins from blood [26]. The purity of EVs isolated from blood plasma could be increased by ultracentrifugation in density gradient [26].

Cerebrospinal fluid yielded roughly 0.1 µg protein in ultracentrifugation pellets from 1 mL of porcine CSF in most samples. For comparison, in the study on human patients with a CSF drain, approximately 2.5 to 5.5 µg of proteins could be obtained in pellets from 1 mL of CSF [47]. The difference in EV protein amount detected in 1 mL of CSF might be due to the EV isolation and analysis techniques, experimental settings (untreated pigs versus patients undergoing thoracic surgery with possible BBB damage and tissue or plasma EV leak to the CSF), or by inter-species variance. 

Seminal plasma yielded not only the largest ultracentrifugation pellets but also the highest amounts of pellet proteins, corresponding on average to 157 µg of proteins obtained from 1 mL of body fluid. Despite the fact that the 100,000 g pellets from seminal plasma may contain not only extracellular vesicles but also large-molecular-weight seminal mucous proteins or protein complexes, the Alix signal in seminal plasma pellets was the strongest from all body fluids studied.

Only small inter-individual variability was observed in 100,000 g pellet protein content in each body fluid among individual samples (Figure 5).

A similar study comparing the EV content in body fluids (milk, blood plasma, saliva, urine) of pregnant dairy cows was recently performed by Koh et al. [53]. The highest numbers of EVs were found in plasma (10^12^ particles per mL), followed by milk. A proteomic analysis revealed a different protein composition of vesicles isolated from milk and plasma, with only eight proteins detected in both body fluid EVs. Saliva and urine (6 mL each) yielded insufficient material for proteomic profiling [53].

## 4. Conclusions

We analyzed the presence of small extracellular vesicles in porcine blood plasma, cerebrospinal fluid, and seminal plasma. In our experimental setting, substantial differences in EV content were identified among body fluids, including up to 1000 times lower protein amount extracted from CSF-derived EVs in comparison to seminal plasma-derived EVs. Blood plasma (1 mL) provides sufficient material for proteomic analyses of EVs in a pig model. However, additional purification/enrichment requiring higher plasma volumes might be necessary for proteomic studies of disease-specific EVs (this was not the subject of this study). EV isolation from CSF for proteomic analyses is complicated by the low EV/protein content. Seminal plasma is a rich source of EVs and in pig it provides sufficient material for EV characterization and proteomic analyses. Only minor inter-individual differences were observed among animals in EV content in particular body fluids.
